# Association between plasma metal exposure and health span in very elderly adults: a prospective cohort study with mixture statistical approach

**DOI:** 10.1186/s12877-024-05001-5

**Published:** 2024-05-01

**Authors:** Xiaoying Ye, Tingting Xu, Le Yang, Xiangju Hu, Xiaowei Xie, Guohui Lan, Xiaoli Lu, Zelin Huang, Tinggui Wang, Jieyu Wu, Jieli Lan, Qian Zhang, Zhiying Zhan, Yansong Guo, Xiaoxu Xie

**Affiliations:** 1 Department of Epidemiology and Health Statistics, School of Public Health, Fujian Medical University, Fuzhou, China; 2 Department for Chronic and Noncommunicable Disease Control and Prevention, Fujian Provincial Center for Disease Control and Prevention, Fuzhou, China; 3 The First Clinical Medical School, Shanxi Medical University, Taiyuan, China; 4 Clinical Research Unit, The Second Affiliated Hospital, Fujian Medical University, Quanzhou, China; 5 Department of Cardiology, Shengli Clinical Medical College of Fujian Medical University, Fujian Provincial Hospital, Fuzhou, China; 6 Fujian Provincial Key Laboratory of Cardiovascular Disease, Fujian Provincial Center for Geriatrics, Fujian Provincial Clinical Research Center for Severe Acute Cardiovascular Diseases, Fuzhou, China; 7 Fujian Heart Failure Center Alliance, Fuzhou, China

**Keywords:** Plasma metal, Mixtures, Health span, Aging, Selenium, Magnesium

## Abstract

**Background:**

Metals have been linked to a diverse spectrum of age-related diseases; however, the effects of metal exposure on health span remains largely unknown. This cohort study aims to determine the association between plasma metal and health span in elder adults aged ≥ 90 years.

**Methods:**

The plasma concentrations of seven metals were measured at baseline in 300 elder adults. The end of the health span (EHS) was identified as the occurrence of one of eight major morbidities or mortality events. We used Cox regression to assess hazard ratios (HR). The combined effects of multiple metal mixtures were estimated using grouped-weighted quantile sum (GWQS), quantile g-computation (Q-gcomp), and Bayesian kernel machine regression (BKMR) methods.

**Results:**

The estimated HR for EHS with an inter-quartile range (IQR) increment for selenium (Se) was 0.826 (95% confidence interval [CI]: 0.737–0.926); magnesium (Mg), 0.806 (95% CI: 0.691–0.941); iron (Fe), 0.756 (95% CI: 0.623–0.917), and copper (Cu), 0.856 (95% CI: 0.750–0.976). The *P* for trend of Se, Mg, and Fe were all < 0.05. In the mixture analyses, Q-gcomp showed a negative correlation with EHS (*P* = 0.904), with the sum of the negative coefficients being -0.211.

**Conclusion:**

Higher plasma Se, Mg, and Fe reduced the risk of premature end of health span, suggesting that essential metal elements played a role in health maintenance in elder adults.

**Supplementary Information:**

The online version contains supplementary material available at 10.1186/s12877-024-05001-5.

## Introduction

In recent years, the global burden of disease in the elder adults has become a major public health concern. In 2019, the main causes of disability-adjusted life-years (DALYs) among people aged ≥ 50 years included ischemic heart disease (IHD), type 2 diabetes, intracerebral hemorrhage, chronic obstructive pulmonary disease (COPD), lung cancer, ischemic stroke, and Alzheimer's disease [1]. In elder adults aged ≥ 70 years and older, the disability-adjusted annual rates for Alzheimer's disease, type 2 diabetes, and lung cancer increased between 1990 and 2019 [1].

Because aging is a common causal influence, common chronic diseases such as heart disease, stroke, cancer, diabetes, kidney disease, and Alzheimer's and Parkinson's disease, chronic diseases that often coexist “comorbidities of ageing” [2]. As individuals age, their cause of death is often attributed to multiple competing factors associated with aging or age-related comorbidities, rather than a single disease. The health span, considered a vital phenotype, is a quantitative and continuous variable that characterizes an individual's ability to age in good health, free from chronic illness or disability. It helps to determine how different genes, environmental parameters, or interventions impact the health span in a quantitative way; it also enables measurement of the relative impact of these factors on the health span rather than the lifespan [3]. Therefore, it is imperative to focus on the influences of environmental exposures on the health span of elder adults; this issue holds significance not only for individuals and families but also for society and the nation as whole.

Magnesium (Mg), calcium (Ca), manganese (Mn), iron (Fe), copper (Cu), zinc (Zn) and other metal elements are crucial for normal biological functions [4]. Its deficiency or excess is related to many disease states of the organism. Among the environmental chemicals to which humans are exposed, these metals play essential roles in the elder adults’ diseases [5–11]. However, the effects of different metals on health span are inconsistent and vary among different studies. A review found that low selenium (Se) levels in the body were associated with shortened lifespan or deteriorating health span in elder adults [12]. Experiments with *Caenorhabditis elegans* have shown that the use of zinc-selective chelators increases the average and maximum lifespans by reducing the level of zinc in the body [13]. In summary, previous studies assessing the effects of metal exposures have either focused on a single metal or on a single outcome, which may have overlooked the potential collinearity and interaction between metals, ignoring the impact of metals on multimorbidity and downplaying their public health significance. Moreover, there is currently limited research on metal exposure and healthy lifespan in population; meanwhile, prospective studies in extremely elder adults are lacking.

Therefore, to fill these knowledge gaps, using data from the China Longitudinal Healthy Longevity Study (CLHLS), a national prospective cohort study in an extremely elder adults, we examined the relationship between seven plasma metals and health span in adults aged ≥ 90 years. This study considered the health span as a result, rather than as a single disease, which is of great significance for healthy aging. Furthermore, we used three models to estimate the combined effects of metal mixture exposure on health span, and comprehensively explained the results, considering the advantages and disadvantages of each method.

### Subjects and methods

#### Study design and participants

**Fig. 1:**
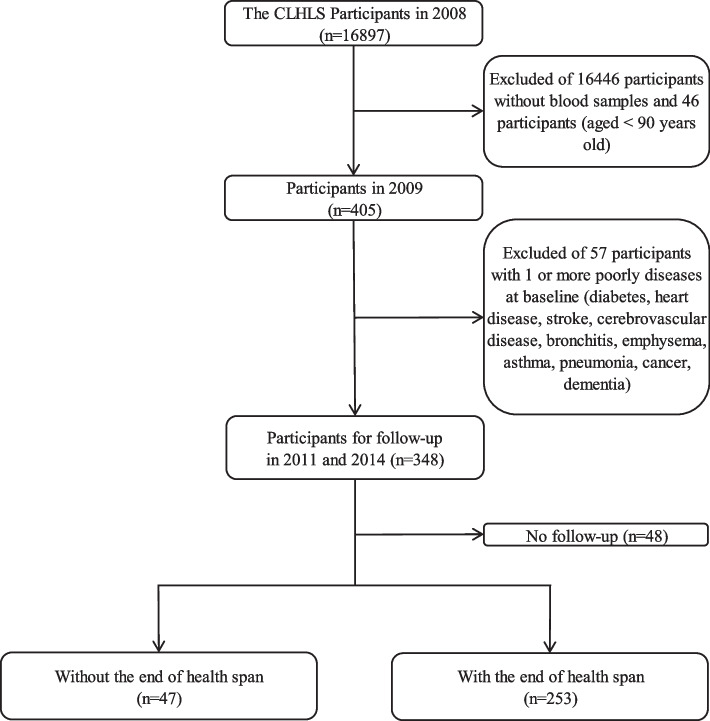
Participant selection flowchart

The study population was drawn from the CLHLS, which is a prospective cohort study conducted in regions known for long-lived populations in China. In 2008, 16897 participants completed a face-to-face questionnaire and underwent comprehensive health examinations; among them, 405 participants aged ≥ 90 years with ID numbers provided blood samples in 2009. At the same time, excluding 57 participants with one or more severely diseases (cerebrovascular disease, stroke, dementia, heart disease, diabetes, pneumonia, asthma, emphysema, bronchitis, or cancer), they conducted two follow-up visits in 2011 and 2014, respectively. Finally, 48 participants were lost to follow-up, 300 elder adults were included in the analysis (Fig. 1). The questionnaire was jointly answered by the respondent and proxy.

#### Ascertainment of the end of health span

The health span is generally defined as aging without loss of healthy function. Among the 293 Global Burden of Diseases causes, 92 (31.4%) were identified as age-related diseases, including cardiovascular diseases, diabetes, neoplasms, Alzheimer's disease, and COPD [14]. Thus, in this study the health span was defined by eight major events (diabetes, heart disease, stroke, cerebrovascular disease, lung diseases, cancer, dementia, or death) that are substantially associated with longevity. The end of the health span was identified as the participant's first occurrence of any of these events during the follow-up.

#### Plasma metal measurement

Plasma concentrations of seven metals—Ca, Cu, Fe, Mg, Mn, Se, and Zn—were measured at baseline. The methods for measuring the seven metals in blood samples were described in previous studies [15] and provided in the Supplementary Method. Fasting venous blood samples were collected and centrifuged using a heparin anticoagulant vacuum tube, and the isolated plasma samples were stored below -20 °C until they could be transported to a central laboratory for metal analysis.

A combination of internal and external calibration was used to correct the measurement bias in plasma metal concentration caused by instrument discrimination. A standard reference material (ClinChek® no. 8882) was used as the control purposes. In the reproducibility test, the metal levels were measured in duplicate for each of the 20 test items, with a relative standard deviation of less than 5% for each metal. Pooled spiked plasma samples were used to assess the precision and accuracy of the method. Recoveries of 90–110% were obtained for each metal.

#### Statistical analyses

The metal concentrations were naturally log-transformed to ensure that the residuals followed a normal distribution when used as continuous variables. Furthermore, the concentrations were categorized into four groups (Q_1_, Q_2_, Q_3_, and Q_4_) based on quartiles and treated as categorical variables. Descriptive analyses of covariates, exposures, and outcomes were performed for the entire study population. Spearman’s correlations were calculated between the logarithmically transformed blood concentrations of the seven different metals.

Firstly, we estimated the hazard ratios (HRs) to assess the impact of metal exposure (quartiles and per one inter-quartile range, IQR) on the end of the health span using a Cox regression model, with the attained age as the time scale. *P* for trends were derived from tests using the median of the metal concentrations within each category. We calculated the *E*-value to evaluate unmeasured confounding factors. The *E*-value was defined as the minimum strength of association on a risk ratio scale that unmeasured confounders needed to have with both the treatment and outcome variables to adequately explain the specific association between treatment and outcome, given the measured covariates. The lowest possible *E*-value is 1, which means that no unmeasured confounders are needed to explain the observed associations. The higher the *E*-value, the stronger the association between the confounders explaining this effect. Secondly, we employed a restricted cubic spline based on Cox regression model to flexibly fit the potential nonlinear relationship between metal concentration (continuous) and the end of the healthy span. *P* for nonlinear was calculated by ANOVA. The model fitted three nodes, with their change points based on the 10th, 50th, and 90th percentiles of metal concentration. Thirdly, we used the quantile g-computation (Q-gcomp) [16] to assess the associations of metal mixtures with health span, which can be based on the Cox model to estimate the risk ratio of quantitative mixing effects. The Q-gcomp results were simple to interpret and computationally convenient, while not assuming directional homogeneity and allowing for nonlinearity and nonadditivity of individual exposure and overall mixing effects.

A series of sensitivity analyses was also performed to demonstrate the robustness and reliability of the results. First, we used the grouped weighted quantile sum (GWQS) model, and Bayesian kernel machine regression (BKMR) as sensitivity analysis for mixture effects [16]. GWQS regression allows the placement of a mixture into groups such that different magnitudes and directions of associations can be determined for each pre-defined group of mixtures. The overall association between metal mixtures and diseases was reported by OR value in GWQS. The BKMR model was used to fit exposure response functions that allowed for nonlinear associations and higher-order interactions, and to calculate conditional posterior inclusion probabilities (condPIP) to determine the relative importance of individual metal exposure, which used 0.5 as the condPIP threshold, to determine whether the metal was significant to the health span [17]. Second, we used the Cox model to conduct a stratified analysis of sex, smoking, alcohol intake, educational level, and body mass index (BMI) to identify susceptible populations. In addition, a multiplication interaction term was included to explore this interaction. Third, we used the inverse probability of weighting (IPW) to control for confounder. IPW is a method that uses observational data to estimate the effects observed in an ideal implementation of a target experiment. This method allows for correction analysis by weighting the observed values, thereby giving them a probability of being selected. Forth, the end of health span was redefined using the leave-one-out method, and then Cox regression was used to estimate the association between each metal and the new outcome, evaluating whether the effect size was heavily influenced by a particular event.

The covariates adjusted for in this study were age, sex, educational level (low or high), smoking status (no or yes), alcohol intake status (no or yes), and BMI. BMI had two missing values that were complemented by the median for the continuous variable. All the statistical analyses were performed using R version 4.1.3. Cox models were constructed using the “survival” package. Exposure–response relationship analyses were performed using the “rms” package. “groupWQS,” “qgcomp,” and “bkmr” packages were used to conduct GWQS, Q-gcomp and BKMR, respectively.

## Results

### Descriptive statistics

The study characteristics are presented in Table 1. A total of 300 participants, including 61 men and 239 women, were included at baseline: 253 participants (84.3%) had the end of health span, 47 participants free of the end of health span. Among the 300 participants, they were born in rural areas (96.0%), had low levels of education (88.0%), and did not smoke (85.3%), or drink (82.7%). Most participants eat meat and fish at least once a week, and rarely take vitamins and medicinal plants (Supplementary Table 1). The average follow-up time in this study was 4 years.

**Table 1:** Description of demographic characterization and plasma metals on the baseline^a^

Characteristic	With the end of health span ( *N* = 253)	Without the end of health span ( *N* = 47)
	Median (P_25_, P_75_) or N (%)	Median (P_25_, P_75_) or N (%)
Age (years)	100.00 (94.00, 102.00)	95.00 (92.00, 100.50)
Sex		
Male	49 (19.4)	12 (25.5)
Female	204 (80.6)	35 (74.5)
Exercise or not at present		
No	216 (85.4)	35 (74.5)
Yes	37 (14.6)	12 (25.5)
Educational level		
Low	228 (90.1)	36 (76.6)
High	25 (9.9)	11 (23.4)
Birthplace		
City	9 (3.6)	3 (6.4)
Rural	244 (96.4)	44 (93.6)
Smoking		
No	213 (84.2)	43 (91.5)
Yes	40 (15.8)	4 (8.5)
Alcohol intake		
No	210 (83.0)	38 (80.9)
Yes	43 (17.0)	9 (19.1)
MMSE^b^	11.0 (0.00, 18.0)	18.0 (11.5, 23.0)
BMI (kg/m^2^)	18.80 (17.22, 20.89)	19.46 (17.57, 21.37)
Selenium (μg/mL)	114.55 (81.79, 151.05)	98.16 (78.60, 128.79)
Manganese (μg/mL)	0.02 (0.01, 0.06)	0.02 (0.01, 0.05)
Magnesium (μg/mL)	23.89 (20.29, 28.08)	24,.34 (20.87, 29.06)
Calcium (μg/mL)	129.00 (102.50, 156.15)	140.92 (103.94, 154.21)
Iron (μg/mL)	3.98 (1.99, 8.19)	4.11 (1.92, 5.56)
Copper (μg/mL)	1.32 (1.06, 1.65)	1.34 (1.04, 1.58)
Zinc (μg/mL)	2.13 (1.10, 3.47)	1.99 (0.87, 3.01)

*Abbreviations: MMSE* Mini-Mental State Examination, *BMI* Body mass index

^a^Continuous variables were expressed as median (quartile1, quartile3), and categorical variables were expressed as numbers (percentages)

^b^The low education group scored ≤ 17 points, with cognitive impairment below the threshold and normal above. The cognitive questionnaire was answered personally by the respondent

**Fig. 2:**
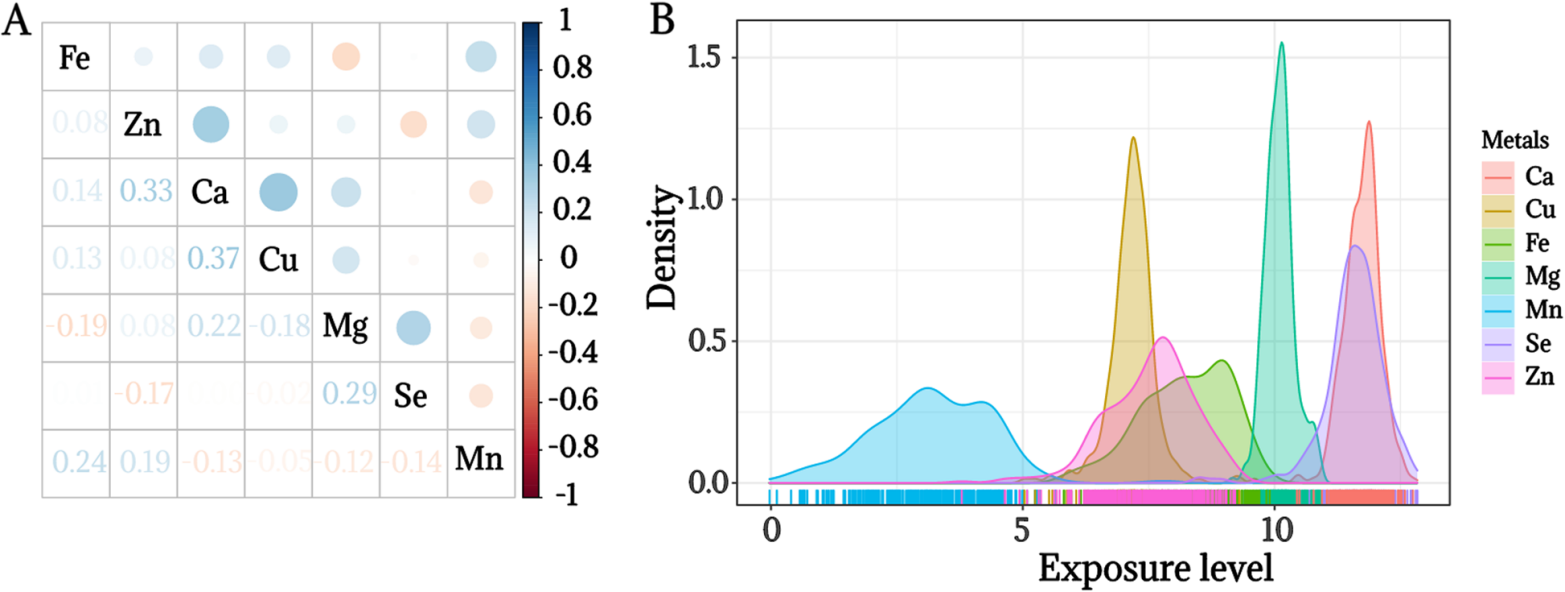
Description of plasma metals. **A** Spearman correlations between plasma metals. **B** Probability density plot between plasma metals. The metal concentrations were natural logarithmically transformed. Abbreviations: Ca Calcium, Cu Copper, Fe Iron, Mg Magnesium, Mn Manganese, Se Selenium, Zn Zinc

Further characterization of the plasma metals showed that the internal correlations between the plasma metals were all weak (*r* < 0.4), with Ca showing the highest correlation with Cu (*r* = 0.37) (Fig. 2A). The metal concentrations of participants ranged from to 0–14 μg/L. The probability density plots of the Cu, Mg, Ca, and Se concentrations showed a more concentrated distribution, whereas the Mn, Zn, and Fe concentrations were more dispersed (Fig. 2B).

### Single metal and health span

The effects of between metal exposure and the end of the health span in older adults are shown in Table 2. In the multivariate model (Model 2), higher Se was associated with a lower risk of ending a healthy life in older people and showed a monotonic downward trend, with an HR of 0.476 (95% confidence interval [CI]: 0.329–0.688) in Q_2_ compared to Q_1_ and an HR of 0.456 (95% CI: 0.318–0.654) in Q_4_ compared to Q_1_. The results for Mg were broadly similar. For Mg (Q_2_ vs. Q_1_), HR: 0.520, 95% CI (0.363–0.745), and (Q_4_ vs. Q_1_), HR: 0.525, 95% CI (0.364–0.755). The trend tests for Se, Mg, and Fe were all statistically significant (*P* < 0.05). In the fourth quartile of Fe, there was a decreasing risk of ending the health span in the elder adults with an HR of 0.571 (95% CI: 0.396–0.825). In addition, Se [HR: 0.826, 95% CI (0.73–0.926)], Mg [HR: 0.806, 95% CI (0.691–0.941)], Fe [HR: 0.756, 95% CI (0.623–0.917)] and Cu [HR: 0.856, 95% CI (0.750–0.976)] were associated with a lower risk of ending the health span for each IQR increment. We calculated the *E*-value for the results of metal exposure as a continuous variable. The results for the *E*-value were in the range of for 1.140–1.980. This suggests that an unmeasured confounder would need to have a risk ratio ≥ 2 with respect to the outcome to fully attenuate the effect values we observed in this study. All of the above results remain consistent in the univariate model (Model 1).

**Table 2:** Associations of plasma metals with the end of health span^a^

plasma metals	Per IQR increment	Quartile 1	Quartile 2	Quartile 3	Quartile 4	*P*-trend	*E*-value^b^
Se							
Model 1	**0.828 (0.727, 0.931)**	1 (reference)	**0.498 (0.345, 0.719)**	0.705 (0.497, 1.000)	**0.498 (0.350, 0.709)**	**0.002**	1.709
Model 2	**0.826 (0.737, 0.926)**	1 (reference)	**0.476 (0.329, 0.688)**	**0.679 (0.477, 0.966)**	**0.456 (0.318, 0.654)**	** < 0.001**	1.716
Mn							
Model 1	1.129 (0.955, 1.334)	1 (reference)	1.153 (0.808, 1.645)	1.210 (0.853, 1.718)	1.296 (0.919, 1.829)	0.129	1.511
Model 2	1.128 (0.949, 1.342)	1 (reference)	1.040 (0.721, 1.500)	1.227 (0.860, 1.750)	1.259 (0.885, 1.790)	0.143	1.508
Mg							
Model 1	**0.828 (0.704, 0.973)**	1 (reference)	**0.507 (0.357, 0.721)**	**0.643 (0.455, 0.911)**	**0.548 (0.384, 0.783)**	**0.010**	1.709
Model 2	**0.806 (0.691, 0.941)**	1 (reference)	**0.520 (0.363, 0.745)**	**0.663 (0.463, 0.949)**	**0.525 (0.364, 0.755)**	**0.005**	1.787
Ca							
Model 1	0.913 (0.787, 1.059)	1 (reference)	0.977 (0.694, 1.375)	0.949 (0.663, 1.359)	0.895 (0.632, 1.268)	0.526	1.418
Model 2	0.941 (0.807, 1.097)	1 (reference)	0.992 (0.703, 1.399)	0.976 (0.677, 1.408)	0.991 (0.695, 1.413)	0.939	1.321
Fe							
Model 1	**0.755 (0.629, 0.907)**	1 (reference)	1.076 (0.761, 1.520)	0.715 (0.499, 1.024)	**0.580 (0.409, 0.823)**	** < 0.001**	1.980
Model 2	**0.756 (0.623, 0.917)**	1 (reference)	1.096 (0.772, 1.556)	0.732 (0.507, 1.056)	**0.571 (0.396, 0.825)**	** < 0.001**	1.976
Cu							
Model 1	**0.870 (0.764, 0.990)**	1 (reference)	0.903 (0.636, 1.281)	0.824 (0.578, 1.174)	0.754 (0.532, 1.070)	0.097	1.564
Model 2	**0.856 (0.750, 0.976)**	1 (reference)	0.906 (0.637, 1.288)	0.733 (0.510, 1.053)	0.736 (0.514, 1.054)	0.058	1.612
Zn							
Model 1	0.953 (0.809, 1.121)	1 (reference)	0.814 (0.571, 1.159)	0.794 (0.556, 1.133)	0.864 (0.610, 1.223)	0.392	1.277
Model 2	0.985 (0.832, 1.167)	1 (reference)	0.932 (0.639, 1.359)	0.864 (0.598, 1.250)	0.950 (0.659, 1.370)	0.704	1.140

Model 1: not adjusted; Model 2: adjusted for sex, BMI, educational level, smoking status, and alcohol intake status

*Abbreviations: IQR* Inter-quartile range, *Ca* Calcium, *Cu* Copper, *Fe* Iron, *Mg* Magnesium, *Mn* Manganese, *Se* Selenium, *Zn* Zinc

^a^Effect estimates were hazard ratios (HRs) and 95%-confidence intervals (95% CIs) derived from the Cox regression model with attained age as the time scale based on the imputed dataset. The metal concentrations were natural logarithmically transformed

^b^The *E*-value was calculated as the result of metals as a continuous variable (per IQR increment)

**Fig. 3:**
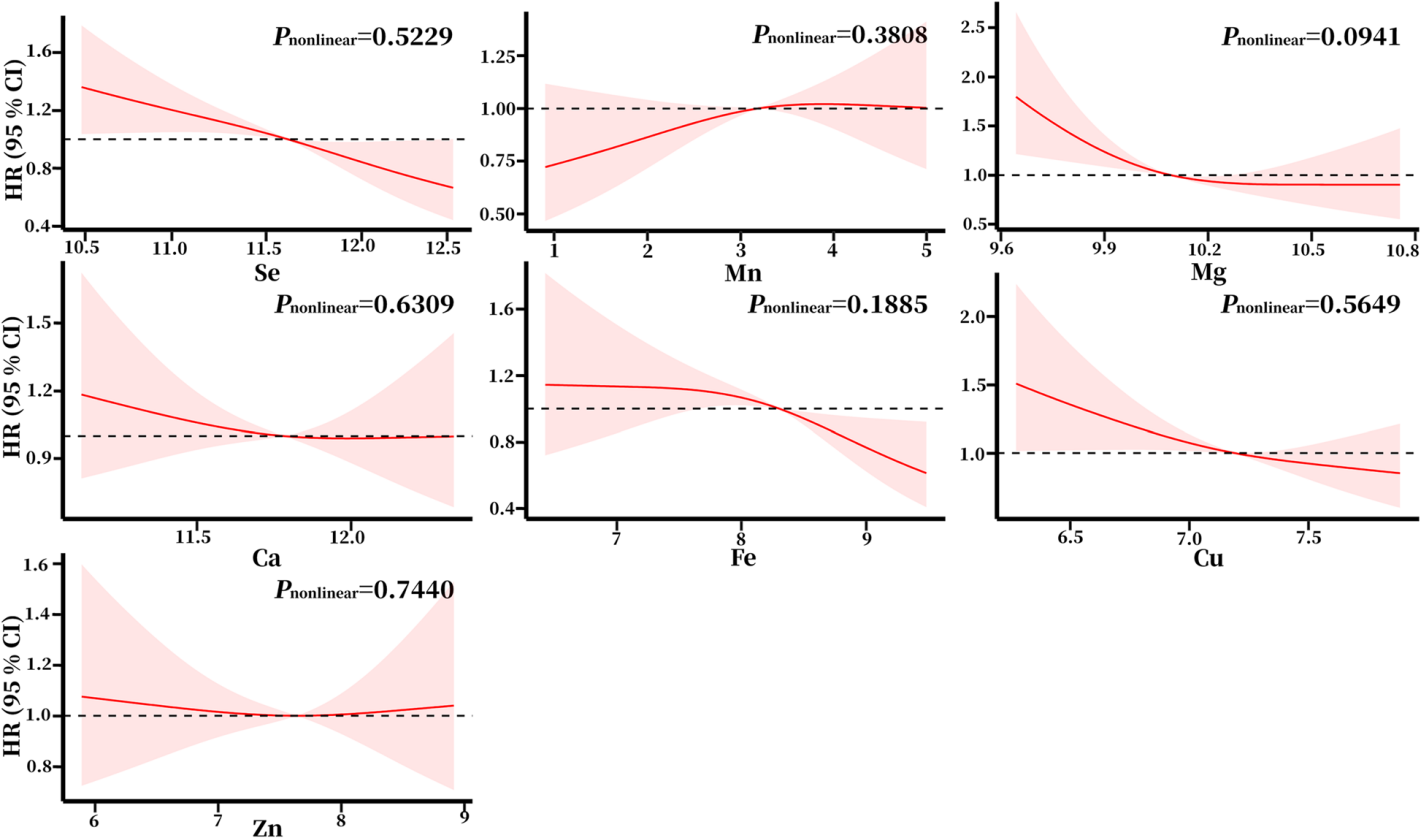
Association between plasma metals and the end of health span based on restricted cubic spline model in aged population. The reference point (HR = 1) was set at the 50th percentage for each metal. The covariates adjusted were sex, BMI, educational level, smoking status, and alcohol intake status. The metal concentrations were natural logarithmically transformed. Abbreviations: HR Hazard ratios, CI Confidence Intervals, Se Selenium, Mn Manganese, Mg Magnesium, Ca Calcium, Fe Iron, Cu Copper, Zn Zinc

The restricted cubic splines showed no significant nonlinear relationship between the end of the health span and the plasma metals (Fig. 3).

### Metal mixtures and health span

**Fig. 4:**
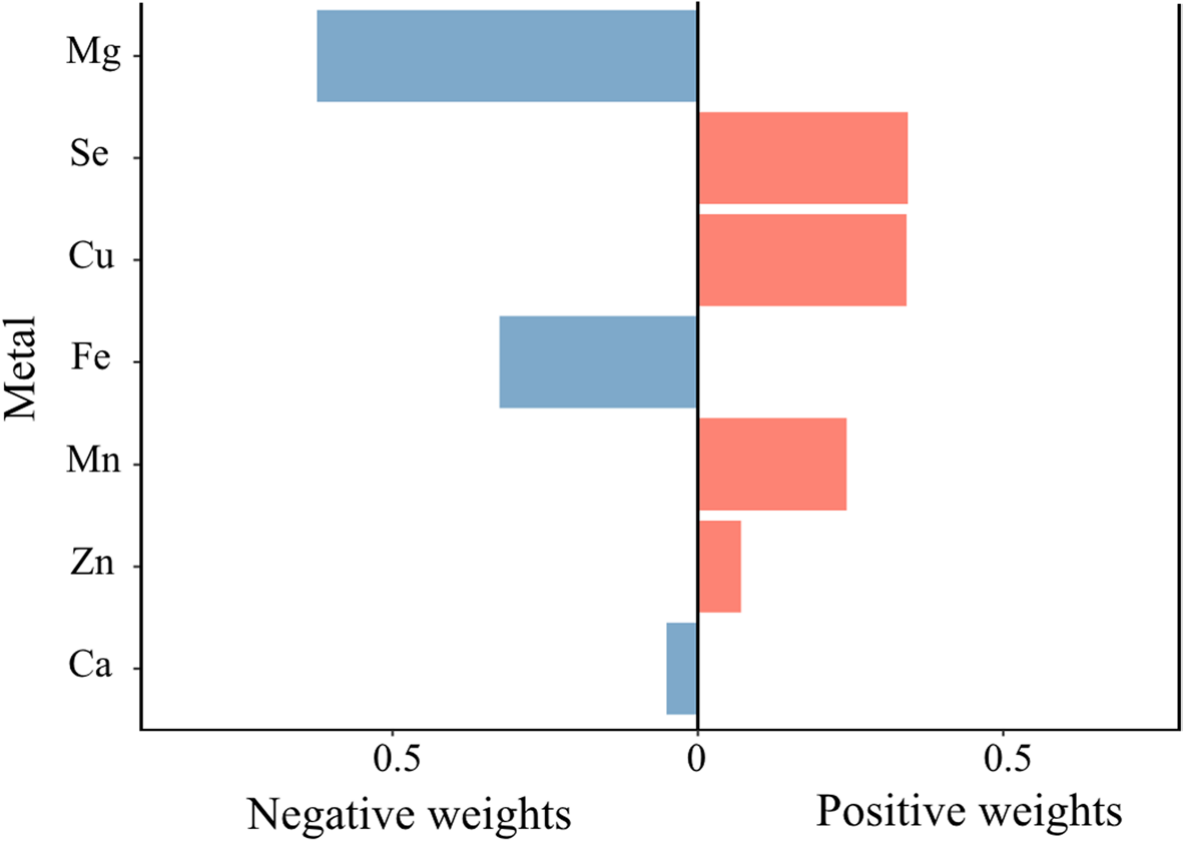
Quantile g-computation and the end of health span. Weight distribution of negative and positive directions of quantile g-computation based on Cox model. The model was adjusted for age, sex, BMI, educational level, smoking status, and alcohol intake status. The metal concentrations were natural logarithmically transformed. Abbreviations: Ca Calcium, Cu Copper, Fe Iron, Mg Magnesium, Mn Manganese, Se Selenium, Zn Zinc

The Q-gcomp analysis based on Cox models, was conducted to assess the combined effect of metal mixture exposures and to identify the metals that contribute to the risk at the end of the health span (Fig. 4). The Q-gcomp regression showed a nonsignificant correlation with the end of the health span (P = 0.904), with the sum of the positive coefficients being 0.193 and the sum of the negative coefficients being -0.211. This suggests that these metals may play an inconsistent role in the association between the metal mixture and health span, and are quite different in driving the observed association.

### Sensitivity analyses

Based on the Cox model and the results of the restricted cubic splines, we divided the metals with an upward trend into the positive direction group and those with a downward trend into the negative direction group for the GWQS analysis. The results showed that both directions were not significant (P > 0.05), with an odds ratio (OR) of 0.976 (95% CI: 0.642–1.481) in the negative direction (Supplementary Fig. 2A) and 1.080 (95% CI: 0.808–1.450) in the positive direction (Supplementary Fig. 2B). The overall effect of the metal mixture showed a moderate but not statistically significant increasing trend (Supplementary Fig. 2C). The BKMR model showed that the condPIP was highest for Cu (0.536), followed by Ca (0.464) (Supplementary Fig. 2E). Based on previous studies [18,19,20], a comparison of the three models was presented in Supplementary Table 10.

Stratified analyses showed a reduced risk of outcomes among those who did not smoke, did not drink alcohol, or had low levels of education (Supplementary Fig. 1). Sex differed in susceptibility to exposure of different metals. Men exposed to Se and Fe had a reduced risk of developing the outcome, whereas women were exposed to Se, Mg and Cu had a decreased risk of developing the outcome. In addition, interactions showed effect modifications between smoking and Mn and Ca, BMI and Mg, and alcohol consumption and Fe. In addition, the inverse probability weighting was applied to the Cox model, and the results remained robust (Supplementary Table 2). Furthermore, after defining the outcome as reducing by one event each time, the results remained robust (Supplementary Tables 3, 4, 5, 6, 7, 8). After excluding death events, there was no association between Mg and the end of the health span, which may be related to a small sample size. But it found a negative correlation between Se and Cu and the end of the health span (Supplementary Table 9).

## Discussion

To the best of our knowledge, very few studies have been published on plasma metals and the health span of the very elder adults. This study aimed to examine the association between plasma metals and the end of the health span in elder adults aged ≥ 90 years residing in a region known for its longevity in China. The main findings of the monometallic model showed that Se, Mg, Fe, and Cu were protective against the end of the health span after controlling for bias. No significant nonlinear associations were observed when restricted cubic splines were used. In the association between metal mixtures and health span, negative direction weighting contributed the most to the quantile g-computation, with the highest weight being Mg (0.624), so the result of GWQS. Additionally, Cu contributed the most to the BKMR model.

A major finding of our study was that higher plasma Se concentrations were associated with a lower risk of ending the health span. Se is an essential micronutrient with various pleiotropic health properties [21]. A nested case–control study demonstrated that plasma levels of Se were negatively associated with hemorrhagic stroke [22]. Furthermore, a mouse trial showed that Se reverses cognitive decline during aging and hippocampal injury [23]. Meanwhile, a trial of Chinese residents aged 40–69 years showed that the consumption of dietary supplements containing 50 μg of Se reduced all-cause and cancer-related mortality [24]. Selenium supplementation inhibits IGF-1 signaling in rats by derepressing the PI3K/AKT/mTOR downstream pro-growth pathway to promote health span [25]. The Etude du Vieillissement Artériel study indicated that lower plasma concentrations of Se were associated with higher mortality in elder adults aged approximately 65 years old [26]. The preventive role of serum Se is further supported by the Women's Health and Ageing Study, which was associated with lower mortality among community-dwelling older women aged 70–79 years [27]. These findings are consistent with our findings. Our findings in the Chinese population reinforce the evidence that higher Se concentrations benefit healthy longevity, which extends the current evidence to the extremely elder adults aged > 90 years.

Our study also found that higher plasma concentrations of Mg might protect against the end of the health span. Magnesium is a crucial mineral involved in regulating approximately 80% of the known cellular and metabolic reactions in the body [28]. Mg deficiency has been linked to a range of chronic diseases, including diabetes, hypertension, heart disease, stroke, dementia, and migraine [29]. Results from a Mendelian randomization study confirmed a consistent association between higher hereditary levels of serum Mg and a decreased incidence of cardiothrombotic stroke [30]. Furthermore, the Hoorn Diabetes Care System cohort demonstrated that the serum Mg^2+^ status was prospectively and inversely related to fatal and nonfatal heart failure and atrial fibrillation [31]. In addition, Mg supplementation can improve the four risk factors for cardiovascular disease [32]. Our results were consistent with these findings. In contrast, treatment of vascular smooth muscle cells from a premature aging mouse model (Lmna G609G/ +) in a magnesium-rich medium increased intracellular ATP levels, enhanced antioxidant capacity, and reduced mitochondrial ROS production, thereby reducing vascular calcification and prolonging mouse lifespan [33].

Our results also revealed that each IQR increase in Fe and Cu levels was associated with a reduced risk of the end of the health span. Fe and Cu are essential minerals and that play pivotal roles in both normal physiological processes and the pathological conditions of various diseases. A Mendelian randomization study indicated that genetically higher Cu levels were linked to a decreased risk of Alzheimer’s disease and bipolar disorder [34]. Iron supplementation can upregulate the mitochondrial tricarboxylic acid cycle and electron transport chain gene expression, and increase ATP levels so as to delay aging and extend the health span [35]. However, several previous studies have reported the detrimental health effects of Fe and Cu. A review found that iron overload was associated with heart disease, and dietary iron overload was also significantly associated with an increased risk of cardiovascular death [36]. Findings on fine particulate air pollution showed that chronic exposure to Fe and Cu in fine particulate matter and their combined effect on reactive oxygen species was correlated with an increase in cardiovascular disease mortality [37]. This relationship remains controversial, which may be owing to differences in the sources of the metals or the characteristics of the study subjects. The metal exposures in this study were endogenous plasma concentrations rather than exogenous exposures to the environment. In addition, the study population included the oldest elder adults, and the limited sample size may not have allowed for a full assessment of the weak association between trace elements and health.

We did not find a statistically significant association between Mn, Zn, Ca, and the health span. Additionally, the results of previous studies have been inconsistent. Ca, Mn, and Zn are essential elements for life, and are present in considerable amounts in the body [38]. A population-based cohort study indicated that high dietary Cu intake was linked to increased cardiovascular disease mortality in both sexes, whereas high dietary Zn intake was associated with lower coronary heart disease mortality in men, but not in women [39]. A review found that zinc deficiency can affect adaptive T-cell immunity, leading to the down-regulation of CD28, and the complete loss of classical auxiliary function (Sub). In addition, this study suggests that zinc supplementation can help restore T cell function in later years and may help alleviate age-related diseases mediated by T-cells [40]. However, it was also found that metal components (Zn and Ca) significantly increased the probability of metabolic syndrome, which is considered as an established risk factor for cardiovascular diseases [41]. Conversely, the results of a meta-analysis suggested that higher Ca intake reduced all-cause mortality [42]. We found that 1,25 (OH)_2_D_3_ deficient mice can significantly reduce oxidative stress, DNA damage, aging cells, and SASP molecular levels in multiple organs by calcium/phosphate supplementation, thereby extending lifespan and improving growth and aging phenotypes [43]. Therefore, more large-scale prospective epidemiological and experimental studies are needed in the future to confirm the causal association between these metals and disease risk and to elucidate the underlying mechanisms.

In this study, we prospectively examined the combined effects of metal mixtures on healthy spans in very elderly adults. Because humans are often exposed to multiple metals simultaneously, it is important to elaborate on the combined effects of multiple metals. High-dimensional collinearity exists between metals, and the health effects of metal mixtures may be antagonistic, synergistic, or additive, which may vary from the effects of single metals. We used Q-gcomp model, as well as the GWQS and BKMR models for sensitivity analysis, but the results were not significant. First, in the Q-gcomp model, some data information may have been lost because of the conversion of continuous exposure variables into quartiles. Second, GWQS and BKMR transformed outcomes into categorical variables not considering survival time, which may lead to heterogeneity in model results. Third, the metals in this study were both positively and negatively correlated and were likely to cancel each other out, causing the mixed model to be insignificant [16]. However, the present evidence on the role of metal mixtures is weak and may seem counterintuitive in the model. Therefore, our findings need to be confirmed and more possibilities should be explored in future studies.

This study had several strengths. First, benefiting from a nationally representative population-based prospective cohort, this study provided prospective evidence of the effects of multiple metals on the health span of older adults. Second, this study considered the effect of metal mixtures, which has often been ignored in previous studies. Furthermore, using the health span as an outcome, rather than a single disease, has important implications for healthy aging. However, there are also some limitations. First, despite of the fact that plasma metals are the most frequently utilized biomarkers, they may not be suitable for evaluating metals with widely fluctuating plasma concentrations and short half-lives and hence may not fully represent dietary supplementation or environmental exposure. Second, the metal concentrations were measured only once at baseline, which limits our ability to capture the long-term exposure status and trajectory of all metals over time. Third, only seven metals (Ca, Cu, Fe, Mg, Mn, Se, and Zn) were included in this study, toxic metals (such as arsenic, cadmium and lead) were not included. Fourth, some subgroups have small sample sizes and stratification analysis results may not be robust.

In conclusion, higher plasma Se and Mg levels were associated with healthy longevity, suggesting a role for these essential trace elements in maintaining health. Future research should elucidate the applicable populations of the current evidence, reveal the biological mechanisms linking these metals to the health span, and explore whether dietary supplements can play a corresponding role.

### Supplementary Information

**Supplementary Material 1. **

## Data Availability

All processed data generated or used during the study appear in the submitted article or supplementary material. The CLHLS data can be requested via the study websites (https://opendata.pku.edu.cn/dataverse/CHADS).
