# Supplementary material for: Association between plasma metal exposure and health span in very elderly adults: a prospective cohort study with mixture statistical approach
Source: BMC Geriatr. 2024 May 1;24:388. doi: 10.1186/s12877-024-05001-5 (PMC11064295; doi:10.1186/s12877-024-05001-5)
Supplement: Supplementary file 1 — Supplementary Material 1. [file 12877_2024_5001_MOESM1_ESM.docx]

**Supplementary Information**

**Association between plasma metal exposure and health span in very elderly adults:**

**a prospective cohort study with mixture statistical approach**

**Supplementary Method.** Plasma metal measurement

**Supplementary Figure 1.** Forest plot of stratification analysis.

**Supplementary Figure 2.** Mixtures of metals and the end of health span.

**Supplementary Table 1.** Description of diet on the baseline.

**Supplementary Table 2.** Associations of plasma metals with the end of health span by using Inverse probability weighting.

**Supplementary Table 3.** Associations between plasma metals and the end of health span without diabetes.

**Supplementary Table 4.** Associations between plasma metals and the end of health span without heart disease.

**Supplementary Table 5.** Associations between plasma metals and the end of health span without stroke, and cerebrovascular disease.

**Supplementary Table 6.** Associations between plasma metals and the end of health span without lung diseases.

**Supplementary Table 7.** Associations between plasma metals and the end of health span without cancer.

**Supplementary Table 8.** Associations between plasma metals and the end of health span without dementia.

**Supplementary Table 9.** Associations between plasma metals and the end of health span without death.

**Supplementary Table 10.** Results summary and model comparison of the three statistical models.

**Supplementary Method.** Plasma metal measurement

For all interviewees, a venous blood sample of 5 ml was collected using a heparin anticoagulated blood collection tube, and blood was collected from the tube for routine blood measurements. Plasma and blood cells were isolated by centrifugation within one hour of collection. Blood samples from heparin anticoagulated tubes are centrifuged at 3000 rpm for 10 minutes at 18-25°C room temperature. It is estimated that 2.4 ml of plasma and 2.6 ml of blood cells can be obtained from 5 ml of whole blood. 1.0-1.2 ml of plasma is taken from the 2.4 ml of plasma into white and blue-capped lyophilization tubes; the middle leukocyte layer is transferred to an orange-colored lyophilization tube, allowing the remaining plasma and lower red blood cells to be added when the leukocytes are removed. Ensure that the codes correspond one to the other. Before the blood sample is transported back to the laboratory for analysis, the sample should be mixed again in the same way as the initial mix.

**Supplementary Figure 1.** Forest plot of stratification analysis. Effect estimates were hazard ratios (HRs) and 95%-confidence intervals (95% CIs) derived from the Cox regression model with attained age as the time scale based on the imputed dataset. The metal concentrations were natural logarithmically transformed. The models were adjusted for sex, BMI, educational level, smoking status, and drinking status. BMI was coded as Categorical variable: 18.50-24.90 kg/m^2^ was coded as normal range, and others were coded as abnormal.

Abbreviations: BMI, body mass index; Ca, Calcium; Cu, Copper; Fe, Iron; Mg, Magnesium; Mn, Manganese; Se, Selenium; Zn, Zinc.

**Supplementary Figure 2.** **Mixtures of metals and the end of health span.** Weight distribution of negative (A) and positive (B) directions grouped weighted quantile sum. (C) Combined metal mixture associations from BKMR model, calculated by comparing the kernel function values when all metals were at specific percentiles (x-axis) with those when all metals were fixed at 50th percentile. (D) Univariate exposure-response associations between individual plasma metal and the end of the health span from BKMR model, where all the other metals were fixed to 50th percentile. (E) BKMR model calculated the conditional posterior inclusion probabilities (condPIP) to identify the relative importance of individual metal exposure, ≥0.5 as threshold for condPIP. All these models were adjusted for age, sex, BMI, educational level, smoking status, and drinking status. The metal concentrations were natural logarithmically transformed. Abbreviations: BKMR, Bayesian kernel machine regression; Ca, Calcium; Cu, Copper; Fe, Iron; Mg, Magnesium; Mn, Manganese; Se, Selenium; Zn, Zinc.

**Supplementary Table 1.** Description of diet on the baseline*.

| Diet | With the end of health span  (N=253) | Without the end of health span  (N=47) | *P*^*^ |
| --- | --- | --- | --- |
|  | N (%) | N (%) |  |
| Meat |  |  | 0.559 |
| ≥ 1 per week | 157 (62.1) | 26 (55.3) |  |
| ≥ 1 per month | 19 (7.5) | 5 (10.6) |  |
| ≥ 1 per year | 77 (30.4) | 16 (34.1) |  |
| Fish |  |  | 0.609 |
| ≥ 1 per week | 111 (43.9) | 17 (36.2) |  |
| ≥ 1 per month | 40 (15.8) | 8 (17.0) |  |
| ≥ 1 per year | 102 (40.3) | 22 (46.8) |  |
| Egg |  |  | 0.333 |
| ≥ 1 per week | 162 (64.0) | 29 (61.7) |  |
| ≥ 1 per month | 39 (15.4) | 11 (23.4) |  |
| ≥ 1 per year | 52 (20.6) | 7 (14.9) |  |
| Vitamins (a/c/e) |  |  | 1.000 |
| ≥ 1 per week | 14 (5.5) | 2 (4.3) |  |
| ≥ 1 per month | 8 (3.2) | 1 (2.1) |  |
| ≥ 1 per year | 231 (91.3) | 44 (93.6) |  |
| Medicinal plants |  |  | 0.051 |
| ≥ 1 per week | 1 (0.4) | 2 (4.3) |  |
| ≥ 1 per month | 2 (0.8) | 1 (2.1) |  |
| ≥ 1 per year | 250 (98.8) | 44 (93.6) |  |

^*^ *P*-value was reported by chi-square test, and the expected count ＜ 5 by Fisher Exact Test.

**Supplementary Table 2.** Associations of plasma metals with the end of health span by using Inverse probability weighting*.

| plasma metals | Per IQR increment | Quartile 1 | Quartile 2 | Quartile 3 | Quartile 4 |
| --- | --- | --- | --- | --- | --- |
| Se | **0.878 (0.784, 0.983)** | 1(reference) | **0.435 (0.278, 0.679)** | **0.650 (0.443, 0.953)** | **0.441 (0.304, 0.641)** |
| Mn | 1.127 (0.932, 1.361) | 1(reference) | 1.028 (0.712, 1.485) | 1.168 (0.820, 1.665) | 1.249 (0.885, 1.761) |
| Mg | **0.815** (0.702, 0.946) | 1(reference) | **0.534 (0.377, 0.755)** | **0.687 (0.490, 0.965)** | **0.571 (0.382, 0.853)** |
| Ca | 0.932 (0.787, 1.105) | 1(reference) | 0.967 (0.686, 1.363) | 0.967 (0.669, 1.399) | 0.976 (0.677, 1.406) |
| Fe | **0.736** (0.600, 0.902) | 1(reference) | 1.118 (0.766, 1.637) | 0.696 (0.483, 1.003) | **0.576 (0.403, 0.824)** |
| Cu | **0.829** (0.714, 0.962) | 1(reference) | 0.905 (0.642, 1.276) | 0.743 (0.520, 1.063) | 0.745 (0.512, 1.084) |
| Zn | 0.986 (0.847, 1.146) | 1(reference) | 0.949 (0.665, 1.354) | 0.818 (0.571, 1.170) | 0.914 (0.636, 1.314) |

*Effect estimates were hazard ratios (HRs) and 95%-confidence intervals (95% CIs) derived from the Cox regression model with attained age as the time scale based on the imputed dataset. The metal concentrations were natural logarithmically transformed. The models were adjusted for sex, BMI, educational level, smoking status, and drinking status.

Abbreviations: IQR, inter-quartile range; Ca, Calcium; Cu, Copper; Fe, Iron; Mg, Magnesium; Mn, Manganese; Se, Selenium; Zn, Zinc.

**Supplementary Table 3.** Associations between plasma metals and the end of health span without diabetes*.

| plasma metals | Per IQR increment | Quartile 1 | Quartile 2 | Quartile 3 | Quartile 4 |
| --- | --- | --- | --- | --- | --- |
| Se | **0.828 (0.739, 0.927)** | 1(reference) | **0.474 (0.328, 0.686)** | **0.694 (0.488, 0.987)** | **0.455 (0.317, 0.653)** |
| Mn | 1.128 (0.948, 1.341) | 1(reference) | 1.056 (0.732, 1.524) | 1.222 (0.857, 1.743) | 1.263 (0.888, 1.795) |
| Mg | **0.799 (0.683, 0.935)** | 1(reference) | **0.519 (0.362, 0.743)** | **0.672 (0.470, 0.963)** | **0.522 (0.362, 0.752)** |
| Ca | 0.938 (0.806, 1.093) | 1(reference) | 1.010 (0.717, 1.424) | 0.964 (0.668, 1.392) | 0.983 (0.690, 1.401) |
| Fe | **0.758 (0.624, 0.920)** | 1(reference) | 1.100 (0.775, 1.561) | 0.731 (0.506, 1.055) | **0.576 (0.398, 0.832)** |
| Cu | **0.853 (0.748, 0.973)** | 1(reference) | 0.917 (0.645, 1.304) | 0.730 (0.508, 1.050) | 0.730 (0.509, 1.045) |
| Zn | 0.986 (0.833, 1.167) | 1(reference) | 0.943 (0.647, 1.376) | 0.862 (0.596, 1.248) | 0.951 (0.659, 1.372) |

*Effect estimates were hazard ratios (HRs) and 95%-confidence intervals (95% CIs) derived from the Cox regression model with attained age as the time scale based on the imputed dataset. The metal concentrations were natural logarithmically transformed. The end of health span included heart disease, stroke, cerebrovascular disease, lung diseases, cancer, dementia, and death. The models were adjusted for sex, BMI, educational level, smoking status, and drinking status.

Abbreviations: IQR, inter-quartile range; Ca, Calcium; Cu, Copper; Fe, Iron; Mg, Magnesium; Mn, Manganese; Se, Selenium; Zn, Zinc.

**Supplementary Table 4.** Associations between plasma metals and the end of health span without heart disease*.

| plasma metals | Per IQR increment | Quartile 1 | Quartile 2 | Quartile 3 | Quartile 4 |
| --- | --- | --- | --- | --- | --- |
| Se | **0.815 (0.729, 0.911)** | 1(reference) | **0.467 (0.322, 0.663)** | **0.654 (0.461, 0.928)** | **0.434 (0.305, 0.618)** |
| Mn | 1.127 (0.952, 1.335) | 1(reference) | 1.139 (0.794, 1.633) | 1.166 (0.820, 1.658) | 1.308 (0.923, 1.856) |
| Mg | **0.819 (0.702, 0.956)** | 1(reference) | **0.550 (0.385, 0.784)** | 0.710 (0.498, 1.011) | **0.553 (0.386, 0.793)** |
| Ca | 0.956 (0.821, 1.113) | 1(reference) | 1.016 (0.722, 1.429) | 0.994 (0.695, 1.423) | 0.999 (0.704, 1.417) |
| Fe | **0.755 (0.624, 0.913)** | 1(reference) | 1.178 (0.831, 1.671) | 0.792 (0.552, 1.136) | **0.590 (0.410, 0.848)** |
| Cu | **0.873 (0.766, 0.994)** | 1(reference) | 0.936 (0.660, 1.327) | 0.786 (0.551, 1.121) | 0.746 (0.524, 1.062) |
| Zn | 0.996 (0.843, 1.177) | 1(reference) | 0.998 (0.684, 1.455) | 0.872 (0.607, 1.251) | 0.991 (0.689, 1.423) |

*Effect estimates were hazard ratios (HRs) and 95%-confidence intervals (95% CIs) derived from the Cox regression model with attained age as the time scale based on the imputed dataset. The metal concentrations were natural logarithmically transformed. The end of health span included diabetes, stroke, cerebrovascular disease, lung diseases, cancer, dementia, and death. The models were adjusted for sex, BMI, educational level, smoking status, and drinking status.

Abbreviations: IQR, inter-quartile range; Ca, Calcium; Cu, Copper; Fe, Iron; Mg, Magnesium; Mn, Manganese; Se, Selenium; Zn, Zinc.

**Supplementary Table 5.** Associations between plasma metals and the end of health span without stroke, and cerebrovascular disease*.

| plasma metals | Per IQR increment | Quartile 1 | Quartile 2 | Quartile 3 | Quartile 4 |
| --- | --- | --- | --- | --- | --- |
| Se | **0.836 (0.744, 0.940)** | 1(reference) | **0.531 (0.368, 0.764)** | 0.714 (0.502, 1.014) | **0.532 (0.373, 0.759)** |
| Mn | 1.170 (0.986, 1.389) | 1(reference) | 0.941 (0.649, 1.363) | 1.327 (0.933, 1.888) | 1.287 (0.907, 1.824) |
| Mg | **0.832 (0.713, 0.971)** | 1(reference) | **0.488 (0.340, 0.702)** | 0.712 (0.499, 1.014) | **0.518 (0.360, 0.745)** |
| Ca | 0.912 (0.785, 1.059) | 1(reference) | 1.029 (0.733, 1.445) | 0.939 (0.650, 1.356) | 0.904 (0.637, 1.283) |
| Fe | **0.764 (0.632, 0.925)** | 1(reference) | 1.077 (0.758, 1.529) | 0.813 (0.566, 1.169) | **0.581 (0.403, 0.835)** |
| Cu | **0.862 (0.754, 0.984)** | 1(reference) | 0.852 (0.599, 1.211) | 0.751 (0.521, 1.081) | 0.757 (0.531, 1.079) |
| Zn | 0.945 (0.803, 1.111) | 1(reference) | 0.929 (0.643, 1.342) | 0.815 (0.566, 1.175) | 0.898 (0.625, 1.291) |

*Effect estimates were hazard ratios (HRs) and 95%-confidence intervals (95% CIs) derived from the Cox regression model with attained age as the time scale based on the imputed dataset. The metal concentrations were natural logarithmically transformed. The end of health span included diabetes, lung diseases, cancer, dementia, and death. The models were adjusted for sex, BMI, educational level, smoking status, and drinking status.

Abbreviations: IQR, inter-quartile range; Ca, Calcium; Cu, Copper; Fe, Iron; Mg, Magnesium; Mn, Manganese; Se, Selenium; Zn, Zinc.

**Supplementary Table 6.** Associations between plasma metals and the end of health span without lung diseases*.

| plasma metals | Per IQR increment | Quartile 1 | Quartile 2 | Quartile 3 | Quartile 4 |
| --- | --- | --- | --- | --- | --- |
| Se | **0.864 (0.761, 0.982)** | 1(reference) | **0.601 (0.407, 0.889)** | 0.724 (0.495, 1.058) | **0.532 (0.361, 0.785)** |
| Mn | 1.139 (0.946, 1.371) | 1(reference) | 0.858 (0.577, 1.275) | 1.111 (0.764, 1.614) | 1.229 (0.852, 1.773) |
| Mg | **0.802 (0.676, 0.951)** | 1(reference) | **0.512 (0.351, 0.746)** | **0.633 (0.433, 0.924)** | **0.517 (0.352, 0.759)** |
| Ca | 0.927 (0.786, 1.092) | 1(reference) | 0.895 (0.623, 1.285) | 0.845 (0.569, 1.256) | 0.951 (0.655, 1.380) |
| Fe | **0.808 (0.656, 0.996)** | 1(reference) | 1.117 (0.763, 1.636) | 0.767 (0.519, 1.133) | **0.675 (0.461, 0.989)** |
| Cu | 0.878 (0.762, 1.011) | 1(reference) | 1.082 (0.742, 1.578) | 0.776 (0.520, 1.159) | 0.827 (0.564, 1.213) |
| Zn | 0.971 (0.817, 1.154) | 1(reference) | 0.999 (0.675, 1.478) | 0.883 (0.599, 1.302) | 0.959 (0.651, 1.412) |

*Effect estimates were hazard ratios (HRs) and 95%-confidence intervals (95% CIs) derived from the Cox regression model with attained age as the time scale based on the imputed dataset. The metal concentrations were natural logarithmically transformed. The end of health span included diabetes, heart disease, stroke, cerebrovascular disease, cancer, dementia, and death. The models were adjusted for sex, BMI, educational level, smoking status, and drinking status.

Abbreviations: IQR, inter-quartile range; Ca, Calcium; Cu, Copper; Fe, Iron; Mg, Magnesium; Mn, Manganese; Se, Selenium; Zn, Zinc.

**Supplementary Table 7.** Associations between plasma metals and the end of health span without cancer*.

| plasma metals | Per IQR increment | Quartile 1 | Quartile 2 | Quartile 3 | Quartile 4 |
| --- | --- | --- | --- | --- | --- |
| Se | **0.832 (0.742, 0.934)** | 1(reference) | **0.473 (0.327, 0.684)** | **0.691 (0.485, 0.984)** | **0.470 (0.328, 0.674)** |
| Mn | 1.144 (0.965, 1.357) | 1(reference) | 1.041 (0.720, 1.504) | 1.313 (0.922, 1.870) | 1.272 (0.896, 1.806) |
| Mg | **0.812 (0.693, 0.951)** | 1(reference) | **0.533 (0.372, 0.763)** | **0.642 (0.448, 0.920）** | **0.538 (0.375, 0.773)** |
| Ca | 0.945 (0.811, 1.101) | 1(reference) | 1.020 (0.724, 1.436) | 1.011 (0.698, 1.463) | 0.981 (0.689, 1.398) |
| Fe | **0.774 (0.640, 0.937)** | 1(reference) | 1.108 (0.780, 1.573) | 0.791 (0.547, 1.143) | **0.602 (0.418, 0.868)** |
| Cu | **0.855 (0.749, 0.974)** | 1(reference) | 0.917 (0.644, 1.304) | 0.758 (0.527, 1.090) | 0.733 (0.512, 1.049) |
| Zn | 0.991 (0.838, 1.171) | 1(reference) | 0.969 (0.667, 1.408) | 0.897 (0.624, 1.291) | 0.959 (0.667, 1.379) |

*Effect estimates were hazard ratios (HRs) and 95%-confidence intervals (95% CIs) derived from the Cox regression model with attained age as the time scale based on the imputed dataset. The metal concentrations were natural logarithmically transformed. The end of health span included diabetes, heart disease, stroke, cerebrovascular disease, lung diseases, dementia, and death. The models were adjusted for sex, BMI, educational level, smoking status, and drinking status.

Abbreviations: IQR, inter-quartile range; Ca, Calcium; Cu, Copper; Fe, Iron; Mg, Magnesium; Mn, Manganese; Se, Selenium; Zn, Zinc.

**Supplementary Table 8.** Associations between plasma metals and the end of health span without dementia*.

| plasma metals | Per IQR increment | Quartile 1 | Quartile 2 | Quartile 3 | Quartile 4 |
| --- | --- | --- | --- | --- | --- |
| Se | **0.822 (0.734, 0.921)** | 1(reference) | **0.501 (0.347, 0.721)** | **0.651 (0.457, 0.929)** | **0.464 (0.325, 0.663)** |
| Mn | 1.135 (0.960, 1.343) | 1(reference) | 1.151 (0.799, 1.658) | 1.356 (0.952, 1.933) | 1.288 (0.906, 1.832) |
| Mg | **0.793 (0.677, 0.929)** | 1(reference) | **0.504 (0.353, 0.719)** | **0.610 (0.428, 0.871)** | **0.491 (0.341, 0.706)** |
| Ca | 0.957 (0.822, 1.115) | 1(reference) | 1.076 (0.764, 1.516) | 1.088 (0.755, 1.567) | 0.995 (0.698, 1.418) |
| Fe | **0.764 (0.631, 0.924)** | 1(reference) | 1.182 (0.835, 1.673) | 0.757 (0.524, 1.093) | **0.627 (0.437, 0.897)** |
| Cu | **0.854 (0.750, 0.973)** | 1(reference) | 0.869 (0.611, 1.237) | 0.753 (0.524, 1.082) | 0.736 (0.516, 1.051) |
| Zn | 0.988 (0.838, 1.166) | 1(reference) | 1.001 (0.695, 1.441) | 0.920 (0.639, 1.324) | 0.960 (0.671, 1.375) |

*Effect estimates were hazard ratios (HRs) and 95%-confidence intervals (95% CIs) derived from the Cox regression model with attained age as the time scale based on the imputed dataset. The metal concentrations were natural logarithmically transformed. The end of health span included diabetes, heart disease, stroke, cerebrovascular disease, lung diseases, cancer, and death. The models were adjusted for sex, BMI, educational level, smoking status, and drinking status.

Abbreviations: IQR, inter-quartile range; Ca, Calcium; Cu, Copper; Fe, Iron; Mg, Magnesium; Mn, Manganese; Se, Selenium; Zn, Zinc.

**Supplementary Table 9.** Associations between plasma metals and the end of health span without death*.

| plasma metals | Per IQR increment | Quartile 1 | Quartile 2 | Quartile 3 | Quartile 4 |
| --- | --- | --- | --- | --- | --- |
| Se | 0.828 (0.616, 1.113) | 1(reference) | **0.236 (0.071, 0.780)** | 0.460 (0.171, 1.234) | **0.256 (0.085, 0.770)** |
| Mn | 1.508 (0.864, 2.632) | 1(reference) | 0.753 (0.208, 2.721) | 0.958 (0.294, 3.123) | 2.424 (0.862, 6.814) |
| Mg | 0.896 (0.563, 1.426) | 1(reference) | 0.721 (0.204, 2.539) | 1.776 (0.588, 5.363) | 0.788 (0.231, 2.687) |
| Ca | **0.678 (0.488, 0.943)** | 1(reference) | 1.116 (0.439, 2.835) | 0.229 (0.047, 1.124) | 0.624 (0.215, 1.815) |
| Fe | 0.860 (0.494, 1.497) | 1(reference) | 0.744 (0.250, 2.208) | 0.363 (0.092, 1.429) | 0.898 (0.330, 2.441) |
| Cu | **0.693 (0.482, 0.997)** | 1(reference) | 2.317 (0.785, 6.837) | 0.943 (0.294, 3.022) | 0.731 (0.218, 2.457) |
| Zn | 1.211 (0.737, 1.989) | 1(reference) | 0.929 (0.280, 3.081) | 1.008 (0.318, 3.190) | 1.390 (0.479, 4.035) |

*Effect estimates were hazard ratios (HRs) and 95%-confidence intervals (95% CIs) derived from the Cox regression model with attained age as the time scale based on the imputed dataset. The metal concentrations were natural logarithmically transformed. The end of health span included diabetes, heart disease, stroke, cerebrovascular disease, lung diseases, cancer, and dementia. The models were adjusted for sex, BMI, educational level, smoking status, and drinking status.

Abbreviations: IQR, inter-quartile range; Ca, Calcium; Cu, Copper; Fe, Iron; Mg, Magnesium; Mn, Manganese; Se, Selenium; Zn, Zinc.

**Supplementary Table 10.** Results summary and model comparison of the three statistical models.

| Model comparison | GWQS | Q-gcomp | BKMR |
| --- | --- | --- | --- |
| Assumption | Data components are divided into Quantiles, which can be reasonably combined into an index and given weight. | It is forced to specify a parameterized nonlinear model. | Continuous outcomes are normally distributed |
| Advantages | GWQS regression allows one to place mixture into groups such that different magnitudes and direction of associations can be determined for each pre-defined group of mixture. | (1) Explains simplicity and computational ease without assuming directional homogeneity; (2) Allows for non-linearity and non-additivity of the effects of individual exposure and the whole mixture. (3) It’s suitable for survival analysis. | (1) Identifies contaminants or groups of contaminants responsible for the observed mixed effects; (2) Enables visualization of exposure-response functions; (3) Detects interactions between individual contaminants. |
| Limitations | (1) Data conversion to quantile may cause loss of data information.  (2) It’s not suitable for survival analysis. | Marginal structure models may not adequately capture the dose response function if the underlying model is not smooth. | (1) The results are inconclusive, and the exploration of the effects of co-exposure to high and low levels of pollutants is limited.  (2) It’s not suitable for survival analysis. |
| Results | Negative direction: Mg (0.260), Se, Ca, Cu, Fe;  Positive direction: Zn (0.562), Mn. | Negative direction: Mg (0.624), Fe, Ca;  Positive direction: Se (0.344), Cu, Mn, Zn. | The condPIP was greatest for Cu (0.536), followed by Ca (0.464). |
| R package | "groupWQS" | "qgcomp" | "bkmr" |

Abbreviations: GWQS, Grouped weighted quantile sum; Q-gcomp, quantile g-computation; BKMR, Bayesian kernel machine regression; Ca, Calcium; Cu, Copper; Fe, Iron; Mg, Magnesium; Mn, Manganese; Se, Selenium; Zn, Zinc.
